# The role of a discussion forum within a web‐based psychoeducational intervention focusing on sex and fertility—What do young adults communicate?

**DOI:** 10.1002/cam4.6317

**Published:** 2023-07-04

**Authors:** Rebecca Skog, Claudia Lampic, Erik Olsson, Lena Wettergren

**Affiliations:** ^1^ Department of Public Health and Caring Sciences Uppsala University Uppsala Sweden; ^2^ Department of Women's and Children's Health Karolinska Institute Stockholm Sweden; ^3^ Department of Psychology Umeå University Umeå Sweden; ^4^ Department of Women's and Children's Health Uppsala University Uppsala Sweden

**Keywords:** breast cancer, clinical cancer research, clinical trials, psychosocial studies, women's cancer

## Abstract

**Objective:**

This study sought to investigate interactive participation and content of a moderated discussion forum within a web‐based psychoeducational intervention aimed at alleviating sexual dysfunction and fertility distress in young adults diagnosed with cancer.

**Methods:**

The study is part of the Fex‐Can Young Adult randomized controlled trial (RCT), in which young adults with self‐reported sexual dysfunction or fertility distress were invited to participate. This study focuses on RCT participants that were randomized into the intervention condition. Sociodemographics and clinical characteristics of intervention participants and level of activity in the intervention were analyzed with descriptive statistics and compared between subgroups (“high” and “low” activity participants). Inductive qualitative thematic analysis was used to analyze the posts in the discussion forum.

**Results:**

Of 135 intervention participants, 24% met the criteria for high activity participation. There were no statistically significant differences found in terms of clinical and sociodemographic characteristics between high and low activity participants. Ninety‐one participants (67%) accessed the discussion forum, and 19 (14%) posted at least once. Posters shared intimate details of their experiences of sexuality and fertility following cancer. The thematic analysis of posts resulted in four themes: fertility fears, perceptions of the changed body, missing out on life, and importance of support and information.

**Conclusions:**

While a smaller proportion of participants posted in the discussion forum, a majority spent time reading posts (lurkers). Participants posting in the forum shared experiences of intimate relationships, body image, parenthood concerns, and support needs. The discussion forum was used by a majority of intervention participants, and provided appreciated support for those who posted in the forum. We therefore recommend similar interventions to include this opportunity for interaction and communication.

## Graphical abstract

This study aimed to investigate interactive participation and content of a moderated discussion forum within a web‐based intervention for young adult cancer survivors experiencing fertility distress and sexual dysfunction. The discussion forum was utilized by a majority of intervention participants, and provided appreciated support for those who posted in the forum. We recommend similar interventions to include this opportunity for interaction and communication among young adult cancer survivors.

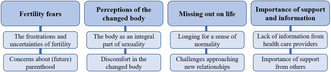

## 1 INTRODUCTION

Worldwide, approximately 1 million young adults (18–39 years) are diagnosed with cancer every year.^1^ Being diagnosed with and treated for cancer may have adverse effects on health‐related quality of life, including sexual and reproductive health.^2^ Previous research has found that approximately 50% of young adults diagnosed with cancer report some degree of sexual dysfunction during the first years post diagnosis,^2^, ^3^ including problems with interest,^4^, ^5^ difficulty reaching orgasm,^4^ and discomfort.^6^ Individuals diagnosed during their reproductive years may further experience concerns about their fertility, with up to 65% of young adult women reporting reproductive concerns following cancer.^7^ These concerns may last long into survivorship,^8^ and includes concerns about fertile ability, health of future children, and fear of not surviving long enough to see potential children grow up.^7^ Given the potential long‐term psychological and physical implications that cancer and its treatment may have on cancer survivors’ sexual and reproductive health, effective and timely interventions are warranted.

Web‐based interventions, as defined by Barak et al., refer to mainly self‐guided, interactive interventions aiming to improve health through delivering education and behavioral change content online.^9^ Such interventions are increasingly being utilized to support individuals with cancer^10^ and have been found effective in reducing various psychological outcomes.^11^, ^12^, ^13^ Few interventions have targeted fertility and sexuality, some showing positive results in reducing reproductive concerns,^14^ and in improving sexual functioning,^15^, ^16^, ^17^ and fertility knowledge.^18^ Inclusion of interactive elements and opportunity for peer discussion in web‐based interventions is recommended as it may increase activity and uptake of interventions,^9^, ^19^ as well as reduce feelings of loneliness.^20^ One study from 2017 assessed the potential effects of including peer support in a web‐based intervention for men with localized prostate cancer.^17^ The intervention was tested in an RCT with three arms: intervention only, intervention plus access to a discussion forum, and access to the forum alone.^17^ It is, to the best of our knowledge, the only study so far that has tested the potential effects of including a discussion forum in a larger web‐based intervention. The study found that participants receiving the intervention in combination with the peer‐led forum reported the greatest improvement, indicating that opportunity to discuss with peers alongside a structured intervention may be beneficial.^17^

Despite the growing number of web‐based interventions, there is limited knowledge about specific interactive elements within interventions.^21^ Additionally, knowledge about how young adults diagnosed with cancer reporting sexual dysfunction and fertility distress use such interventions is lacking. Thus, this study sought to investigate interactive participation and content of a moderated discussion forum included in a web‐based intervention aimed at alleviating sexual dysfunction and fertility distress in young adults diagnosed with cancer.

## 2 METHODS

### 2.1 Study design

This study is part of the Fex‐Can (Fertility and Sexuality following Cancer) Young Adult project, which includes a national cohort study investigating fertility concerns and sexual dysfunction the first 5 years following a cancer diagnosis during young adulthood (Fex‐Can Cohort)^22^ with an embedded randomized controlled trial (Fex‐Can RCT).^23^ Cohort participants rating fertility distress and/or sexual dysfunction at predefined levels were invited to participate in an RCT testing the effects of a web‐based psychoeducational intervention, delivered in two programs: Fex‐Can Fertility and Fex‐Can Sexuality.^23^ Efficacy of the Fex‐Can Fertility program have been reported previously,^18^ and analysis of the Fex‐Can Sexuality program is currently ongoing. This study concerns a subset of the Fex‐Can RCT participants (i.e., those randomized to receive either of the intervention programs) and investigates interactive participation and content of a moderated discussion forum. Posts written in the forum were analyzed using qualitative thematic analysis. The intervention is reported in line with the Template for Intervention Description and replication (TIDier) checklist.^24^

The trial was registered in the ISRCTN registry, January 25, 2016 (ISRCTN36621459).

### 2.2 Participants

All young adults (18‐39 years) diagnosed with selected cancers (breast, cervical, ovarian, testicular cancer, brain tumors, and lymphoma) during a period of 18 months were identified in national quality registries and invited to participate in the Fex‐Can Cohort study approximately 1.5 years following diagnosis.^22^ Individuals reporting fertility distress or sexual dysfunction at the baseline assessment of the Fex‐Can Cohort study were eligible for participation in the RCT and subsequently invited to the study.^23^

**FIGURE 1:**
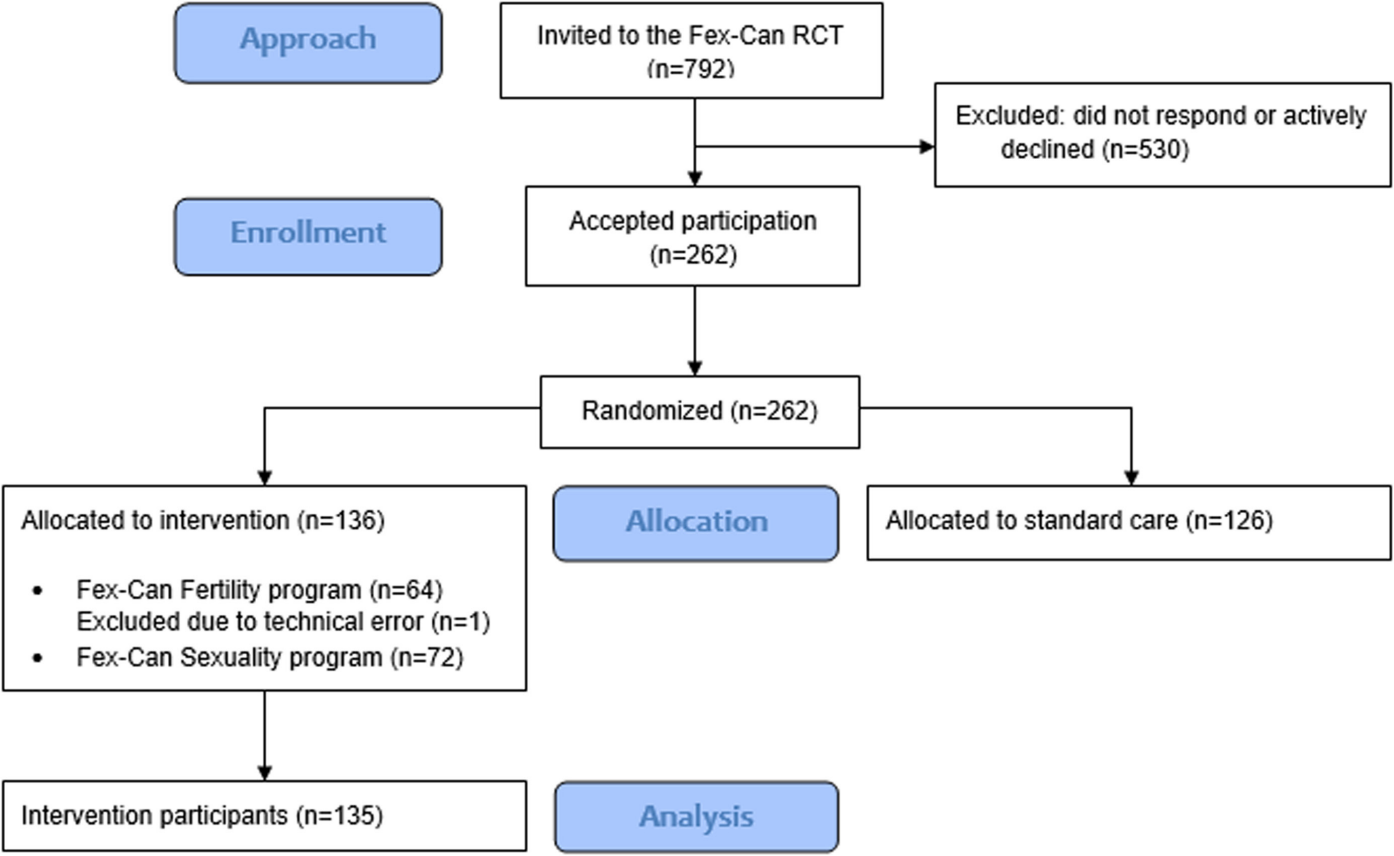
Flow diagram of study participants. RCT, randomized controlled trial.

Among those invited to the intervention (*n* = 792), 262 consented and were randomized to intervention group (*n* = 136) or control group (*n* = 126), see Figure 1. Sixty‐four intervention group participants were invited to the Fertility program, and 72 to the Sexuality Program. Due to a technical error, one participant did not receive the allocated intervention, resulting in a final sample of 135 participants. This study presents data from intervention group participants of the Fex‐Can RCT.

### 2.3 Psychoeducational intervention

The web‐based psychoeducational intervention was developed in collaboration with patient research partners and consisted of two separate programs: Fex‐Can Fertility and Fex‐Can Sexuality, targeting fertility and sexuality, respectively.^23^ The intervention was delivered in six consecutive models during 12 weeks, with a new module being introduced every other week (Table 1).

**TABLE 1:** Overview of modules included in the fertility and sexuality programs.

***Fex‐Can Fertility***	**Shared Discussion forum**	***Fex‐Can Sexuality***
Fertility following cancer		Sexuality
Handling distress		Lack of desire
Trying to achieve a pregnancy		Discomfort and pain (females) Erection (males)
Own and child's health		Orgasm (females) Orgasm and ejaculation (males)
Not able to have biological children		Relationships and sex
Relationships		My body

The intervention was designed to facilitate satisfaction of participants' basic needs (competence, relatedness, and autonomy) according to self‐determination theory (SDT).^25^ It consisted of educational and behavior change content, exercises, photos and illustrations, short videos with young adult cancer survivors, and interactive quizzes and open‐ended questions.^23^ Further, the intervention included an asynchronous moderated discussion forum available for joint discussion between programs throughout the intervention period, and was included to facilitate the SDT‐component relatedness.^25^ In the discussion forum, participants could respond to predefined topics posted by the moderators, to posts made by other participants, or just read without writing posts of their own (so‐called “lurking”).^26^ Participants were able to be anonymous by using an alias of their choice. The forum was moderated by one of the patient research partners, and by members of the research team with clinical expertise in psychology or nursing, providing additional questions and encouraging information. The feasibility of the intervention has been assessed with satisfying results.^27^

### 2.4 Data collection

Sociodemographics (country of birth, parenting children, education, occupation, partnership status, and sexual orientation) and various patient‐reported outcomes, including instruments to assess sexual function and fertility distress were collected through a questionnaire completed approximately 1.5 years post diagnosis. Age and clinical data (diagnosis, treatment) were retrieved from the National cancer quality registers for the included diagnoses.

Data on intervention activity was retrieved from the intervention portal and included, for example, time spent in modules and discussion forum, quizzes and reflective questions responded to, and posts written in the discussion forum.

### 2.5 Data analysis

Based on intervention activity, participants were categorized into high or low activity participants. This categorization has previously been used in a study testing the efficacy of the Fex‐Can Fertility program.^18^ Definition of high activity was based on: opening >50% of intervention modules and spending ≥20 min on the website, plus one of the following: (a) spending ≥3 min in the discussion forum, (b) answering ≥50% of the quizzes and reflective questions, and (c) posting ≥1 post in the discussion forum. Participants who did not fulfill these criteria were low activity participants, including those who never logged on to the intervention. Possible differences between subgroups (“high activity” vs “low activity”) were tested using Student's *t*‐test (age), chi‐square test, and Fisher's exact tests. All tests were two‐tailed with *p* < 0.05 indicating statistical significance. The analyses were conducted using SPSS Statistics for Windows, version 28 (IBM Corp.).

Posts in the discussion forum were analyzed using inductive qualitative thematic analysis as described by Braun and Clarke.^28^ After initial familiarization of the material, including reading and rereading the text material and note‐making, coding was conducted by the first author (RS). Following coding of the entire text material, potential themes, and subthemes were constructed and subsequently reviewed. Codes, themes, and subthemes were discussed among co‐authors in an iterative process to ensure that they accurately captured the content of discussion forum posts. The posts were further categorized based on type of post^29^: describing own experiences, relating or responding to posts written by others, or asking for the experiences of others.

### 2.6 Ethical considerations

Ethical approval for the Fex‐Can Cohort and RCT study was obtained by the Regional Ethical Review Board in Stockholm, Sweden (Dnr: 2013/1746‐31/4; 2014/2244‐32;2015/2042‐32/4; 2017/916‐32; 2017/1416‐32).

Individuals eligible for participation in the RCT received an information letter about the intervention and study procedures via postal mail. The letter further contained information about the voluntary nature of participation, and that participants could withdraw their consent at any time. Written informed consent was obtained from all participants. Data were processed according to the EU General Data Protection regulation (GDPR).

## 3 RESULTS

### 3.1 Participant characteristics

Sociodemographics and clinical characteristics of participants in the RCT are presented in Table 2 for the total group, subgroups demonstrating low/high activity level, and additionally for the group posting messages in the discussion forum (posters). The majority were women, and mean age at study start was 33.6 years (SD: 5.3, range: 20–41). The most common diagnosis for participants was breast cancer (44%) followed by cervical cancer (19%), and lymphoma (15%). Mean age at diagnosis was 32.4 years (SD: 5.3, range: 19–39).

**TABLE 2:** Sociodemographics and clinical characteristics of RCT participants by level of activity and writing posts in the discussion forum (“posters”).

	Total *n* = 135	Activity categorization of total group		Posters *n* = 19
	*n* (%)	Low activity *n* = 102	High activity *n* = 33	
		*n* (%)	*n* (%)	*n* (%)
Sociodemographics				
Sex				
Female	110 (81)	82 (80)	28 (85)	17 (89)
Male	25 (19)	20 (20)	5 (15)	2 (11)
Fex‐Can program				
Sexuality	72 (53)	56 (55)	16 (48)	7 (37)
Fertility	63 (47)	46 (45)	17 (52)	12 (63)
Age at study entry, years				
Mean (SD)	33.6 (5.31)	33.8 (5.59)	32.9 (4.34)	31.6 (4.18)
Country of birth				
Sweden	118 (87)	88 (86)	30 (91)	18 (95)
Other country	17 (13)	14 (14)	3 (9)	1 (5)
Educational level				
University	89 (66)	66 (65)	23 (70)	11 (58)
Upper secondary	37 (27)	30 (29)	7 (21)	4 (21)
Elementary	1 (1)	‐	1 (3)	‐
Other	8 (6)	6 (6)	2 (6)	4 (21)
Occupation				
Working/studying	101 (75)	78 (76)	23 (70)	15 (79)
Unemployed, sick leave, and other^a^	34 (25)	24 (24)	10 (30)	4 (21)
Sexual orientation				
Heterosexual	123 (91)	93 (91)	30 (91)	15 (79)
Nonheterosexual	12 (9)	9 (9)	3 (9)	4 (21)
Partner				
Partnered	102 (76)	77 (75)	25 (76)	15 (79)
Non‐partnered	33 (24)	25 (25)	8 (24)	4 (21)
Children				
Yes	75 (56)	58 (57)	17 (52)	7 (37)
No	60 (44)	44 (43)	16 (48)	12 (63)
Clinical characteristics				
Type of cancer				
Breast cancer	59 (44)	44 (43)	15 (45)	7 (37)
Cervical cancer	26 (19)	18 (18)	8 (24)	2 (11)
Ovarian cancer	4 (3)	4 (4)	‐	1 (5)
Brain tumor	13 (10)	10 (10)	3 (9)	3 (16)
Lymphoma	20 (15)	17 (17)	3 (9)	4 (21)
Testicular cancer	13 (10)	9 (9)	4 (12)	2 (11)
Treatment intensity^b^				
Least intensive or extensive	20 (15)	16 (16)	5 (16)	3 (17)
Moderately intensive or extensive	35 (27)	26 (26)	9 (29)	6 (33)
Very intensive or extensive	73 (56)	56 (55)	17 (55)	9 (50)
Most intensive or extensive	3 (2)	3 (3)	‐	‐

*Note*: All comparisons between the low and the high activity group were nonsignificant (*p* > 0.05).

^a^ Other: parental leave, other.

^b^ According to the adapted version of the intensity treatment rating scale.^30^

### 3.2 Intervention activity

One hundred and twenty‐four out of the 135 participants (92%) logged on to the intervention website, 65/135 participants (48%) opened at least half of the intervention modules, and 42% spent at least 20 min on the website. The majority (*n* = 102, 76%) were classified as low activity participants. Comparison of the subgroups “high activity” and “low activity” showed no statistically significant differences with regard to sociodemographic and clinical characteristics (data not shown).

### 3.3 Discussion forum

Ninety‐one (67%) participants accessed the discussion forum, spending a mean time of 7.9 min (SD 21.0). While most participants accessing the discussion forum lurked rather than wrote own post, 19 (14%) participants wrote a total of 57 posts in the forum.

**FIGURE 2:**
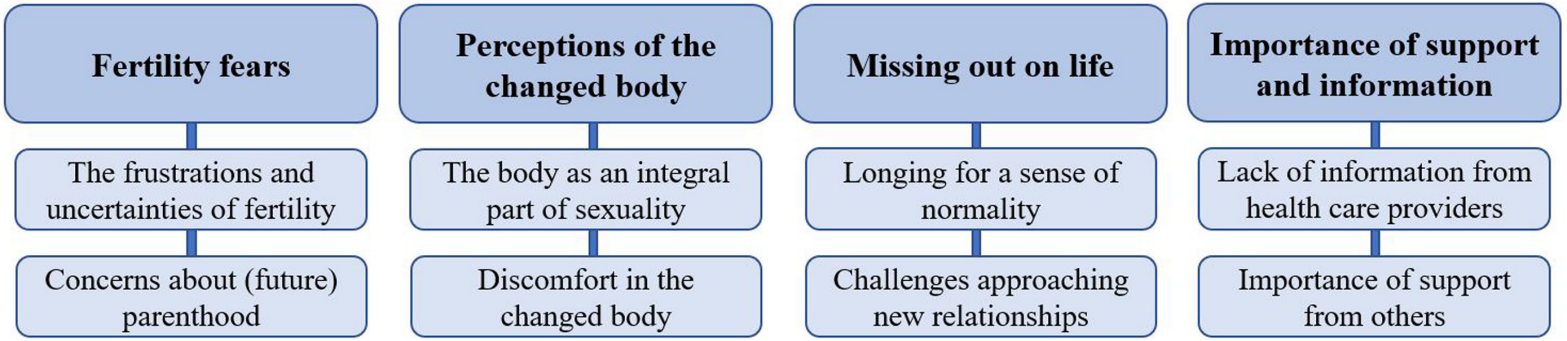
Themes and subthemes from the thematic analysis of the discussion forum.

Most posts were descriptions of own experiences (no. of posts = 33; 58%), and about a third (no. of posts = 19; 33%) related, or was a response, to posts made by others. Five (9%) posts asked for the experiences of others. The thematic analysis of the posts resulted in four main themes: *fertility fears*, *perceptions of the changed body*, *missing out on life*, and *importance of support and information* (Figure 2).

#### 3.3.1 Fertility fears

##### The frustrations and uncertainties of fertility

The importance of fertility was heightened following diagnosis, causing concerns and frustrations for both parous and nulliparous participants. Among those with children some described that, although being content with number of children before diagnosis, it was difficult to accept that the decision was no longer theirs.

Me and my husband had decided that we were not going to have any more children, we were both fine with that decision, but, when I got cancer and the doctor told me that part of the treatment meant that I would be “chemically castrated” I started to contemplate over potentially having another child. It was like a protest in my brain – nobody is going to come here and decide whether I should have more children or not. (Woman, 28 years old).

Concerns about fertility were further enhanced in relation to (potential) partners, particularly for participants without children. Wanting to give partners children, but not knowing whether that would be possible, resulted in feelings of stress and guilt. The two male posters described concerns that, due to their potential fertility problems, their female partners may have to go through burdensome fertility treatments.

##### Concerns about (future) parenthood

Concerns about fertility changed over time. Initial fears of not being able to have children were combined with concerns about future parenthood, causing them to fear both infertility and having children simultaneously.

Honestly, I started to think about how my disease will affect my future children. There's the risk of cancer recurrence, and that I might have to go through another brain surgery in about 15‐20 years…How do you prepare your child for a reality where their mother won't be a good mother? (Woman, 27 years old).

#### 3.3.2 Perceptions of the changed body

##### Discomfort with bodily changes

Cancer and its treatment caused uncomfortable changes to participants' bodies. Mastectomy, weight gain or loss, and scars caused many to feel dissatisfied with their appearance.

I cried when I first saw myself in the mirror. I had a scar that went from the side of the face up behind the hair line…I felt like Frankenstein's monster every time I saw myself in the mirror. (Woman, 27 years old).

Changes in appearance was particularly distressing when it came to perceptions of others. Some felt uncomfortable even leaving the house, while others described discomfort being naked in front of partners. While some avoided putting themselves in potentially uncomfortable situations completely, others tried to conceal their bodies by wearing particular clothing, or by covering their face with pillows during sexual intimacy. Comments, or even fear of potential comments, about their bodies made various social activities all the more intimidating.

Another concern brought up was changes in functioning, often attributed to fatigue post‐treatment. This was described both in relation to very specific concerns, for example, about how fatigue and reduced ability to multitask stopped them from participating in various activities, as well as in more general sense of feeling let down by their bodies, causing frustration for many.

##### The body as an integral part of sexuality

Changes to the body further affected participants' sexuality, and in turn, their thoughts and feelings about intimacy. For some, these changes were mainly about their changed function and sexual sensations.

Now, after a while, it is not at all what is was like before, and it will probably never be like that again. I feel like the whole desire aspect is dependent on other factors now. If I am tired, it is almost impossible [to have sex], and similarly with other feelings. It is like my body no longer has the hormones that creates that sudden urge. (Man, 32 years old).

For others, changes in appearance reduced their interest in sexual activities. By not feeling comfortable in their bodies, they described not feeling attractive in front of their partners, and thus uncomfortable with sexual activity. Contradicting the other participants, one woman described how her being diagnosed with a life‐threatening disease made her more accepting of her appearance and thus took greater pleasure in her sexuality.

#### 3.3.3 Missing out on life

##### Longing for a sense of normality

Feeling closed off from others, and the everyday life, was repeatedly brought up in the forum. Participants described sorrows and concerns, as well as frustrations over their experienced inability to live as they used to.

I feel like my cancer closed me off, in a social way, I felt like all of a sudden it was really difficult to socialize in the same pace as before. The life of others just keeps on going as usual, while I feel like mine was completely ruined by cancer, it deprived me of being personal and open, committed and social. (Woman, 29 years old).

##### Challenges approaching new relationships

Dating and approaching new relationships was not only a particularly challenging aspect following diagnosis and treatment, but also something that many longed for. Participants who had met their partners during or after treatment, or were currently dating, described struggling with how to communicate with their partners about problems experienced following cancer. Talking about sexuality with new partners seemed to be particularly difficult, due to fear of rejection, or feelings of guilt toward partners.

I was single before my treatment and I still am today. There's a clear difference before and after treatment when it comes to sexuality…To explain this to partners, and particularly in a new relationship can feel intimidating. I feel like there is an expectation on me as a man to really seek out and almost hunt intercourse and the physical rather than the intimacy. (Man, 33 years old).

#### 3.3.4 Importance of support and information

##### Lack of information from health‐care providers

Many participants described not having received any information about sexuality or fertility from their health‐care providers. Rather, they had to either ask for information, or look for it on their own. Although having a lot of questions, they did not know who to talk to.

Nobody told me that it could affect my chances to have children…I recently found out that the cancer is on its way back too, so I am wondering if it's not better to hurry up. I don't know who to talk to about this though. I think there is a lack of information about this with cancer, sex, and fertility in general. (Woman, 24 years old).

##### Importance of support from others

Emotional support from loved ones, and having someone to talk to, was of great importance to participants. The importance further became all the more apparent when experiencing a lack of it. Participants struggled finding individuals to talk to, particularly in regard to fertility and sexuality. Feeling that people did not understand them caused them to feel closed off from others. Posting in the discussion forum, and thus being able to communicate with individuals who they perceived as understanding, was something many felt very grateful for.

I've always struggled with talking about my sexuality, and I am even more uncomfortable now. I haven't had anyone to talk to about this, so I feel incredibly grateful to take part of this study, and to have people to talk to who understands. (Woman, 35 years old).

## 4 DISCUSSION

This study investigated interactive participation and content of a moderated discussion forum within a web‐based intervention aimed at alleviating sexual dysfunction and fertility distress among individuals diagnosed with cancer during young adulthood. The majority (76%) of the participants were not very active in the intervention (low activity participants). Two thirds accessed the discussion forum, and a smaller proportion (14%) wrote own posts sharing intimate experiences of fertility and sexuality. The thematic analysis resulted in four main themes: *fertility fears*, *perceptions of the changed body*, *missing out on life*, and *importance of support and information*.

Overall activity in the intervention appeared to be low, with about a fourth fulfilling criteria for high activity participation. High activity participants did not differ statistically significantly regarding sociodemographic and clinical characteristics compared to the rest of the intervention group. The proportion of participants that posted in the discussion forum appear to be in line with a report of use of an online infertility peer support forum^31^ as well as similar study directed to young adults following childhood cancer.^29^ Spending time in forums without posting, so‐called “lurking,” defined as “anyone who reads but seldom if ever publicly contributes to an online group,”^26^ is common in online support groups.^32^, ^33^ Posters and lurkers do not seem to differ in terms of sociodemographic variables,^34^ but previous studies indicate that posters may have more psychological distress than lurkers and nonusers.^31^ Although lurking is a legitimate way of participating, there must be a number of posters for the forum to be beneficial. Previous research has recommended ways to increase activity in discussion forums, for example, by having moderators,^35^ by contacting participants prior to the start of the intervention,^35^ and by providing encouraging information and predefined discussion topics.^33^, ^35^

Posters in this study shared a variety of intimate details of their experiences of sexuality and fertility following cancer, including concerns about body image, romantic relationships and future parenthood, in line with what has been reported in previous studies.^36^, ^37^ Posters further described receiving little information about such concerns from their health‐care providers, and experienced difficulties in finding someone to talk to. Participants expressed taking comfort in reading others' posts, and appreciated the support that their peers provided them. Those who described not having their support needs met in their everyday lives felt particularly grateful for the opportunity to discuss and connect with peers in the discussion forum. Opportunity to be anonymous, and the presence of moderators may have played a role in facilitating a trusting and comfortable environment, enabling participants to share intimate aspects of their lives in the forum.^38^

Participants described feeing uneasy discussing sexuality and fertility with (potential) partners, in line with previous studies.^7^, ^39^ For young adults with cancer, communicating about and disclosing problems with sexuality and fertility may be particularly uncomfortable, since many may be in new relationships or dating.^37^ Concerns about fertility was experienced both in regard to fertile ability but also in regard to ability to be adequate parents to (future) children, consistent with previous research.^36^ In this study, fertility concerns were experienced by participants with and without children prior to diagnosis, and regardless of them knowing whether their fertile ability was impaired or not. Bodily changes caused many participants great distress and discomfort, and participants described how this affected their everyday lives, including (potential) relationships and sexuality. Young adults with cancer frequently report dissatisfaction with their bodies following cancer and treatment, and how this dissatisfaction in turn affect their sexuality.^40^ In this study, the body was discussed separately in terms of appearance (e.g., scars, weight gain/loss) and function (e.g., fatigue, changed sensations, and ways of being), with both experienced as having negative effects on sexuality, social activities, and overall perceived ability to live life like before their cancer diagnosis.

### 4.1 Limitations

This study comes with a number of strengths and limitations. A strength of this study is that participants were drawn from a large sample of a national cohort of individuals diagnosed with cancer during young adulthood.^22^ This provides equal opportunity for young men and women diagnosed with six different cancer types, and experiencing fertility distress or sexual problems, to participate in the intervention. Few men participated in the intervention, and few posted in the discussion forum, making it hard to draw conclusions for male participants. Additionally, few individuals born outside of Sweden participated, limiting the generalizability of study results. The availability of data on clinical and sociodemographic variables for all intervention participants allowed for analysis of potential influencing factors on level of activity in the intervention. However, the criteria for activity level was defined a posteriori^18^ and whether they correctly distinguish level of activity in the intervention is unclear. The subgroup analyses had low power, but based on the similar proportions of characteristics, it is unlikely that larger samples would have resulted in significant group differences.

### 4.2 Clinical implications

An Internet‐delivered peer discussion forum appears to be suitable for young adults to share and communicate experiences of fertility and sexuality following cancer. Participants posting in the forum expressed appreciation for the support received from peers and moderators. Additionally, the relatively high proportion of participants “lurking” in the forum indicates that the discussion forum is used by a majority of participants. Further, no negative accounts of discussion forum participation were reported, indicating that those who posted in the forum felt comfortable engaging with peers and moderators.

## 5 CONCLUSION

This study adds to the limited previous research on interactive participation in web‐based interventions. In line with previous reports, a smaller proportion of participants posted in the discussion forum, while a majority spent time reading posts (lurkers). Participants posting in the forum shared experiences of intimate relationships, body image, parenthood concerns, and support needs. The discussion forum was utilized by a majority of intervention participants and appreciated by discussion forum posters. We therefore recommend similar interventions to include this opportunity for interaction and communication among young adult cancer survivors.

## AUTHOR CONTRIBUTIONS

**Rebecca Skog:** Conceptualization (equal); formal analysis (lead); methodology (equal); project administration (supporting); validation (equal); visualization (lead); writing – original draft (lead); writing – review and editing (equal). **Claudia Lampic:** Conceptualization (equal); data curation (lead); formal analysis (equal); funding acquisition (lead); investigation (lead); methodology (lead); project administration (lead); resources (lead); validation (equal); visualization (supporting); writing – review and editing (equal). **Erik Olsson:** Conceptualization (supporting); formal analysis (supporting); methodology (supporting); supervision (supporting); validation (supporting); visualization (supporting); writing – review and editing (equal). **Lena Wettergren:** Conceptualization (equal); data curation (lead); formal analysis (equal); funding acquisition (lead); investigation (lead); methodology (lead); project administration (lead); resources (lead); supervision (lead); validation (equal); visualization (supporting); writing – original draft (supporting); writing – review and editing (equal).

## FUNDING INFORMATION

This study was supported by grants from The Swedish Cancer Society (CAN 2013/886, CAN 2016/615, 190196Pj), the Cancer Research Funds of Radiumhemmet (161272), the Swedish Research Council for Health, Working Life and Welfare (2014‐4689, 2019‐00839) the Swedish Research Council (2017‐01530), and the Vårdal Foundation (2014‐0098).

## ETHICS STATEMENT

Ethical approval has been obtained for the study procedures by the Swedish Ethical Review Authority (Dnr: 2013/1746‐31/4; 2014/2244‐32; 2017/916‐32; 2017/1416‐32).

## CLINICAL TRIAL REGISTRATION NUMBER

The trial was registered in the ISRCTN registry, January 25, 2016 (ISRCTN36621459).

## Supporting information


Table S1.

## Data Availability

The data that support the findings of this study are available from the last author [LW], upon reasonable request.

## References

[1] Fidler MM , Gupta S , Soerjomataram I , Ferlay J , Steliarova‐Foucher E , Bray F . Cancer incidence and mortality among young adults aged 20–39 years worldwide in 2012: a population‐based study. Lancet Oncol. 2017;18(12):1579‐1589. doi:10.1016/S1470-2045(17)30677-0 29111259

[2] Acquati C , Zebrack BJ , Faul AC , et al. Sexual functioning among young adult cancer patients: a 2‐year longitudinal study. Cancer. 2018;124(2):398‐405. doi:10.1002/cncr.31030 29149503 PMC7514897

[3] Wettergren L , Kent EE , Mitchell SA , et al. Cancer negatively impacts on sexual function in adolescents and young adults: the AYA HOPE study. Psychooncology. 2017;26(10):1632‐1639. doi:10.1002/pon.4181 27240019 PMC7239373

[4] Wettergren L , Eriksson LE , Bergström C , et al. Prevalence and risk factors for sexual dysfunction in young women following a cancer diagnosis—a population‐based study. Acta Oncol. 2022;61(10):1165‐1172. doi:10.1080/0284186X.2022.2112283 36176069

[5] Ljungman L , Eriksson LE , Flynn KE , et al. Sexual dysfunction and reproductive concerns in young men diagnosed with testicular cancer: an observational study. J Sex Med. 2019;16(7):1049‐1059. doi:10.1016/j.jsxm.2019.05.005 31255211

[6] Ljungman L , Ahlgren J , Petersson LM , et al. Sexual dysfunction and reproductive concerns in young women with breast cancer: type, prevalence, and predictors of problems. Psychooncology. 2018;27(12):2770‐2777. doi:10.1002/pon.4886 30203884 PMC6585728

[7] Gorman JR , Su HI , Roberts SC , Dominick SA , Malcarne VL . Experiencing reproductive concerns as a female cancer survivor is associated with depression. Cancer. 2015;121(6):935‐942. doi:10.1002/cncr.29133 25377593 PMC4352116

[8] Ussher JM , Perz J , Miller A , et al. Threat of biographical disruption: the gendered construction and experience of infertility following cancer for women and men. BMC Cancer. 2018;18(1):250. doi:10.1186/s12885-018-4172-5 29506492 PMC5836444

[9] Barak A , Klein B , Proudfoot J . Defining internet‐supported therapeutic interventions. Ann Behav Med. 2009;1(38):4‐17. doi:10.1007/s12160-009-9130-7 19787305

[10] Viola A , Panigrahi G , Devine KA . Digital interventions for adolescent and young adult cancer survivors. Curr Opin Support Palliat Care. 2020;14(1):51‐59. doi:10.1097/SPC.0000000000000480 31842020

[11] van den Berg SW , Gielissen MFM , Custers JAE , van der Graaf WTA , Ottevanger PB , Prins JB . BREATH: web‐based self‐management for psychological adjustment after primary breast cancer—results of a multicenter randomized controlled trial. J Clin Oncol. 2015;33(25):2763‐2771. doi:10.1200/JCO.2013.54.9386 26169621

[12] Yun YH , Lee KS , Kim YW , et al. Web‐based tailored education program for disease‐free cancer survivors with cancer‐related fatigue: a randomized controlled trial. J Clin Oncol. 2012;30(12):1296‐1303. doi:10.1200/JCO.2011.37.2979 22412149

[13] Winzelberg AJ , Classen C , Alpers GW , et al. Evaluation of an internet support group for women with primary breast cancer. Cancer. 2003;97(5):1164‐1173. doi:10.1002/cncr.11174 12599221

[14] Su HI , Stark S , Kwan B , et al. Efficacy of a web‐based women's health survivorship care plan for young breast cancer survivors: a randomized controlled trial. Breast Cancer Res Treat. 2019;176(3):579‐589. doi:10.1007/s10549-019-05260-6 31054032 PMC6626763

[15] Hummel SB , van Lankveld JJDM , Oldenburg HSA , et al. Efficacy of internet‐based cognitive behavioral therapy in improving sexual functioning of breast cancer survivors: results of a randomized controlled trial. J Clin Oncol. 2017;35(12):1328‐1340. doi:10.1200/JCO.2016.69.6021 28240966

[16] Schover LR , Strollo S , Stein K , Fallon E , Smith T . Effectiveness trial of an online self‐help intervention for sexual problems after cancer. J Sex Marital Ther. 2020;46(6):576‐588. doi:10.1080/0092623X.2020.1762813 32400321

[17] Wootten AC , Meyer D , Abbott JAM , et al. An online psychological intervention can improve the sexual satisfaction of men following treatment for localized prostate cancer: outcomes of a Randomised Controlled Trial evaluating My Road Ahead. Psychooncology. 2017;26(7):975‐981. doi:10.1002/pon.4244 27503036

[18] Micaux C , Wiklander M , Eriksson LE , Wettergren L , Lampic C . Efficacy of a web‐based psychoeducational intervention for young adults with fertility‐related distress following cancer (Fex‐Can): randomized controlled trial. JMIR Cancer. 2022;8(1):e33239. doi:10.2196/33239 35348459 PMC9006131

[19] Brouwer W , Kroeze W , Crutzen R , et al. Which intervention characteristics are related to more exposure to internet‐delivered healthy lifestyle promotion interventions? A systematic review. J Med Internet Res. 2011;13(1):e2. doi:10.2196/jmir.1639 21212045 PMC3221341

[20] Ussher J , Kirsten L , Butow P , Sandoval M . What do cancer support groups provide which other supportive relationships do not? The experience of peer support groups for people with cancer. Soc Sci Med. 2006;62(10):2565‐2576. doi:10.1016/j.socscimed.2005.10.034 16303220

[21] Wootten AC , Pillay B , Abbott JAM . Can sexual outcomes be enhanced after cancer using online technology? Curr Opin Support Palliat Care. 2016;10(1):81‐86. doi:10.1097/SPC.0000000000000189 26730795

[22] Wettergren L , Ljungman L , Micaux Obol C , Eriksson LE , Lampic C . Sexual dysfunction and fertility‐related distress in young adults with cancer over 5 years following diagnosis: study protocol of the Fex‐Can Cohort study. BMC Cancer. 2020;20(1):722. doi:10.1186/s12885-020-07175-8 32758179 PMC7409491

[23] Lampic C , Ljungman L , Micaux Obol C , Eriksson LE , Wettergren L . A web‐based psycho‐educational intervention (Fex‐Can) targeting sexual dysfunction and fertility‐related distress in young adults with cancer: study protocol of a randomized controlled trial. BMC Cancer. 2019;19(1):344. doi:10.1186/s12885-019-5518-3 30975116 PMC6458789

[24] Hoffmann TC , Glasziou PP , Boutron I , et al. Better reporting of interventions: template for intervention description and replication (TIDieR) checklist and guide. BMJ. 2014;7(348):g1687. doi:10.1136/bmj.g1687 24609605

[25] Ryan RM , Deci EL . Self‐determination theory and the facilitation of intrinsic motivation, social development, and well‐being. Am Psychol. 2000;55:68‐78. doi:10.1037/0003-066X.55.1.68 11392867

[26] Nonnecke B , Preece J . Silent participants: Getting to know lurkers better. In: Lueg C , Fisher D , eds. From Usenet to CoWebs: Interacting with Social Information Spaces. Springer‐Verlag; 2003.

[27] Wiklander M , Strandquist J , Obol CM , et al. Feasibility of a self‐help web‐based intervention targeting young cancer patients with sexual problems and fertility distress. Support Care Cancer. 2017;25(12):3675‐3682. doi:10.1007/s00520-017-3793-6 28721554 PMC5658457

[28] Braun V , Clarke V . Thematic analysis. APA Handbook of Research Methods in Psychology. Research designs: Quantitative, qualitative, neuropsychological, and biological. Vol 2. American Psychological Association; 2012:57‐71. (APA Handbooks in Psychology®). doi:10.1037/13620-004

[29] Gottvall M , Fagerkvist K , Lampic C , Wettergren L . Including a discussion forum in a web‐based intervention on fertility and sexuality following cancer—usage and content. Internet Interv. 2022;29:100559. doi:10.1016/j.invent.2022.100559 35845087 PMC9284441

[30] Hedman C , Ahlgren J , Smedby KE , et al. Cancer in young adulthood—classifying the intensity of treatment. Acta Oncol. 2022;61(7):809‐813. doi:10.1080/0284186X.2022.2071110 35575147

[31] O'Connell SBL , Gelgoot EN , Grunberg PH , et al. ‘I felt less alone knowing I could contribute to the forum’: psychological distress and use of an online infertility peer support forum. Health Psychol Behav Med. 2021;9(1):128‐148. doi:10.1080/21642850.2021.1884556 34104553 PMC8158233

[32] Preece J , Nonnecke B , Andrews D . The top five reasons for lurking: improving community experiences for everyone. Comput Hum Behav. 2004;20(2):201‐223. doi:10.1016/j.chb.2003.10.015

[33] Sun N , Rau PPL , Ma L . Understanding lurkers in online communities: a literature review. Comput Hum Behav. 2014;1(38):110‐117. doi:10.1016/j.chb.2014.05.022

[34] Klemm P . Effects of online support group format (moderated vs peer‐led) on depressive symptoms and extent of participation in women with breast cancer. Comput Inform Nurs. 2012;30(1):9‐18. doi:10.1097/NCN.0b013e3182343efa 22240564

[35] Classen CC , Chivers ML , Urowitz S , et al. Psychosexual distress in women with gynecologic cancer: a feasibility study of an online support group. Psychooncology. 2013;22(4):930‐935. doi:10.1002/pon.3058 22374732

[36] Gorman JR , Bailey S , Pierce JP , Su HI . How do you feel about fertility and parenthood? The voices of young female cancer survivors. J Cancer Surviv. 2012;6(2):200‐209. doi:10.1007/s11764-011-0211-9 22179785 PMC3667153

[37] Shaw LK , Sherman K , Fitness J . Dating concerns among women with breast cancer or with genetic breast cancer susceptibility: a review and meta‐synthesis. Health Psychol Rev. 2015;9(4):491‐505. doi:10.1080/17437199.2015.1084891 26315681

[38] Wiljer D , Urowitz S , Barbera L , et al. A qualitative study of an internet‐based support group for women with sexual distress due to gynecologic cancer. J Cancer Educ. 2011;26(3):451‐458. doi:10.1007/s13187-011-0215-1 21594587

[39] Gorman JR , Smith E , Drizin JH , Lyons KS , Harvey SM . Navigating sexual health in cancer survivorship: a dyadic perspective. Support Care Cancer. 2020;28(11):5429‐5439. doi:10.1007/s00520-020-05396-y 32157507

[40] Cherven B , Sampson A , Bober SL , et al. Sexual health among adolescent and young adult cancer survivors: a scoping review from the Children's Oncology Group Adolescent and Young Adult Oncology Discipline Committee. CA Cancer J Clin. 2021;71(3):250‐263. doi:10.3322/caac.21655 33283888 PMC8678924

