# Supplementary material for: The role of a discussion forum within a web‐based psychoeducational intervention focusing on sex and fertility—What do young adults communicate?
Source: Cancer Med. 2023 Jul 4;12(16):17273–83. doi: 10.1002/cam4.6317 (PMC10501254; doi:10.1002/cam4.6317)
Supplement: Supplementary file 1 — Table S1. [file CAM4-12-17273-s001.docx]

| **Supplementary table.** Sociodemographics and clinical characteristics of RCT participants by level of activity with P-values | | | | |
| --- | --- | --- | --- | --- |
|  | Total  n=135 | Activity categorization of total group | |  |
|  |  | Low activity  n=102 | High activity  n=33 |  |
|  | n (%) | n (%) | n (%) | P-value |
| **Sociodemographics** | | | |  |
| *Sex*  ^a^ Female  Male | 110 (81)  25 (19) | 82 (80)  20 (20) | 28 (85)  5 (15) | .57 |
| *Fex-Can Program*  ^a^ Sexuality  Fertility | 72 (53)  63 (47) | 56 (55)  46 (45) | 16 (48)  17 (52) | .52 |
| *Age at study entry, years* ^c^  Mean (SD) | 33.6 (5.31) | 33.8 (5.59) | 32.9 (4.34) | .41 |
| *Country of birth* ^b^  Sweden  Other country | 118 (87)  17 (13) | 88 (86)  14 (14) | 30 (91)  3 (9) | .49 |
| *Educational level* ^a^  University  Non-university ^d^ | 89 (66)  46 (34) | 66 (65)  36 (35) | 23 (70)  10 (30) | .60 |
| *Occupation* ^a^  Working/studying  Unemployed, sick leave, other ^e^ | 101 (75)  34 (25) | 78 (76)  24 (24) | 23 (70)  10 (30) | .44 |
| *Sexual Orientation* ^b^  Heterosexual  Non-heterosexual | 123 (91)  12 (9) | 93 (91)  9 (9) | 30 (91)  3 (9) | .96 |
| *Partner* ^a^  Partnered  Non-partnered | 102 (76)  33 (24) | 77 (75)  25 (25) | 25 (76)  8 (24) | .98 |
| *Children* ^a^  Yes  No | 75 (56)  60 (44) | 58 (57)  44 (43) | 17 (52)  16 (48) | .59 |
| **Clinical characteristics** | | | | |
| *Type of Cancer* ^b^  Breast cancer  Cervical cancer  Ovarian cancer  Brain tumor  Lymphoma  Testicular cancer | 59 (44)  26 (19)  4 (3)  13 (10)  20 (15)  13 (10) | 44 (43)  18 (18)  4 (4)  10 (10)  17 (17)  9 (9) | 15 (45)  8 (24)  -  3 (9)  3 (9)  4 (12) | .68 |
| *Treatment Intensity* ^b^*^,^* ^f^  Least intensive or extensive  Moderately intensive or extensive  Very intensive or extensive  Most intensive or extensive | 20 (15)  35 (27)  73 (56)  3 (2) | 16 (16)  26 (26)  56 (55)  3 (3) | 5 (16)  9 (29)  17 (55)  - | .79 |
| Numbers do not sum up due to missing data  ^a^ Tested by Chi-square test ^b^ Tested by Fisher’s exact test  ^c^ Tested by Student’s t-test  ^d^ Elementary school, upper secondary school, or other education  ^e^ Other: parental leave, other ^f^ According to the adapted version of the intensity treatment rating scale. | | | | |
